# Preparation and Optoelectronic Characteristics of ZnO/CuO-Cu_2_O Complex Inverse Heterostructure with GaP Buffer for Solar Cell Applications

**DOI:** 10.3390/ma6104479

**Published:** 2013-10-09

**Authors:** Chih-Hung Hsu, Lung-Chien Chen, Yi-Feng Lin

**Affiliations:** Department of Electro-optical Engineering, National Taipei University of Technology, 1, sec.3, Chung-Hsiao E. Rd., Taipei 106, Taiwan; E-Mails: bluerex6@ms35.hinet.net (C.H.H.); lovemor0811@hotmail.com (Y.F.L.)

**Keywords:** CuO whiskers, Cu_2_O, gallium phosphide, energy gap widen effect

## Abstract

This study reports the optoelectronic characteristics of ZnO/GaP buffer/CuO-Cu_2_O complex (COC) inverse heterostructure for solar cell applications. The GaP and COC layers were used as buffer and absorber in the cell structure, respectively. An energy gap widening effect and CuO whiskers were observed as the copper (Cu) layer was exerted under heat treatment for oxidation at 500 °C for 10 min, and arose from the center of the Cu_2_O rods. For preparation of the 30 nm-thick GaP buffer by sputtering from GaP target, as the nitrogen gas flow rate increased from 0 to 2 sccm, the transmittance edge of the spectra demonstrated a blueshift form 2.24 to 3.25 eV. Therefore, the layer can be either GaP, GaNP, or GaN by changing the flow rate of nitrogen gas.

## 1. Introduction

Cuprous oxide (Cu_2_O) is a potential material to replace traditional silicon and other semiconductors, being favorable because of the fabrication of cheaper solar cells for terrestrial applications, its non-toxic nature, the abundance of the starting material (copper), and cheap production processing [1,2,3,4,5]. The Cu_2_O is intrinsically p-type, and has an energy gap (Eg) of 2.17 eV [6,7,8]. The p-type nitrogen-doped Cu_2_O (Cu_2_O:N) has an Eg of 2.5 eV owing to the energy gap widening effect [9]. Several articles have reported on Cu_2_O/transparent conductive oxide structure solar cells [8,10,11,12]. However, the efficiency of the Cu_2_O-based solar cells is far below the maximum theoretical efficiency of 20% [7,8]. Poor minority carrier transport is a factor limiting the performance of Cu_2_O-based solar cells [13,14,15]. Therefore, it has to introduce a buffer layer to improve the carrier collection and to optimize the cells performance. The buffer layer is a layer situated the absorber and the transparent conductive oxide. It should satisfy the following conditions: (1) a thin layer; (2) energy gap between the absorber and the transparent conductive oxide; and (3) lattice constant between the absorber and the transparent conductive oxide [16].

Gallium phosphide (GaP) is an indirect semiconductor and the band gap is ~2.26 eV [17,18,19]. The energy gap of GaP is between the absorber (CuO-Cu_2_O) and the transparent conductive oxide (ZnO). Therefore, this study employed GaP with ~2.26 eV absorption edge as a buffer layer in Cu_2_O-based solar cell. This article reports on the characteristics of Ga(N)P layers grown using magnetron reactive sputtering deposition using a GaP target. In addition, this work address a structure consisting of ZnO/GaP buffer/CuO-Cu_2_O complex (COC) with cascade band gap. The fabrication and optoelectronic performance of a ZnO/GaP buffer/COC inverse heterostructure solar cell are also considered.

## 2. Experimental Section

**Figure 1:**
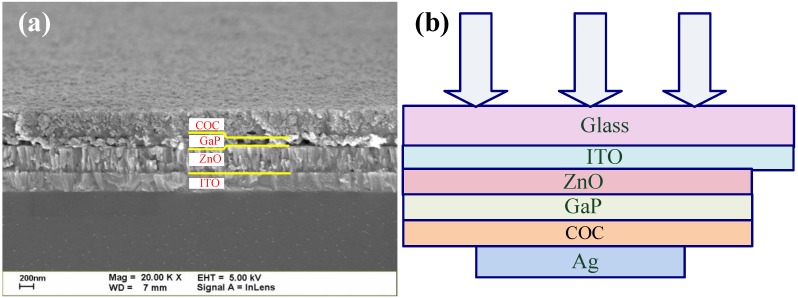
(**a**) Cross-sectional scanning electron microscopy (SEM) image of the ZnO/GaP/COC heterostructure; (**b**) Schematic cross section of the completed structure.

Firstly, zinc oxide (ZnO) layers were formed on an indium tin oxide-coated glass (ITO glass) substrate by magnetron reactive sputtering from ZnO targets in argon (Ar) gas at a flow rate of 15 sccm and a stable pressure of 3 × 10^−3^ Torr. Next, gallium (nitride) phosphide [a(N)P] layers were deposited onto the ZnO layer by sputtering from GaP single crystal targets in nitrogen gas at a flow rate of 0–2 sccm and Ar gas at a flow rate of 40 sccm. Sequently, one micron thick copper (Cu) layers were deposited with a purity of 99.995% onto the Ga(N)P films by sputtering from Cu targets in Ar gas at a flow of 15 sccm. The CuO-Cu_2_O complex (COC) films were obtained by oxidation of Cu layers annealing at various temperatures for 10 min in air ambient in a furnace. Finally, an Ag electrode was formed by sputtering onto the surface of the COC layer to complete Ag/COC/GaNP/ZnO/ITO inverse heterostructure solar cells. Figure 1 shows the cross sectional field-emission scanning electron microscopy (FESEM) images and the completed structure.

All films were prepared on glass in order to measure their electronic characteristics by Hall measurements and their absorbance and transmittance spectra. The film crystalline was studied by X-ray diffraction (XRD). An He-Cd laser (325 nm) was used as the excitation source for taking the photoluminescence (PL) measurements. Additionally, the current density-voltage (J-V) characteristics were determined using a Keithley 2420 programmable source meter under irradiation by a 100 W xenon lamp. Finally, the irradiation power density on the surface of the sample was calibrated as 100 W/m^2^ in order to prevent thermal radiation damaging the samples because the xenon lamp operating at 1000 W/m^2^ is very hot.

## 3. Results and Discussion

**Figure 2:**
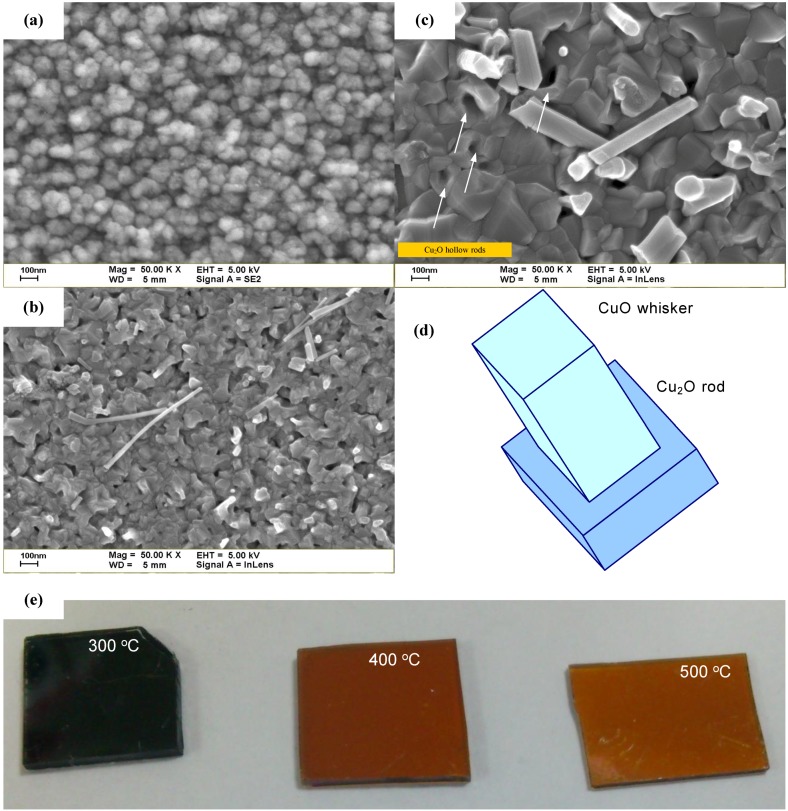
(**a**–**c**) SEM images of the samples with thermal oxidation at various temperatures; (**d**) Schematic of CuO whiskers; (**e**) Optical microscopy images of the samples with thermal oxidation at various temperatures.

Figure 2 shows the FESEM images of the COC layers with thermal oxidation at various temperatures. The image of the COC layer with thermal treatment at 300 °C indicates that the surface of the film consisted of small particles. The average particle size was approximately 100 nm, as shown in Figure 2a. The crystal grains of COC layer were formed as oxidation temperature increase up to 400 and 500 °C, and CuO whiskers were observed, as shown in Figure 2b,c [20,21,22]. The CuO whiskers were extended from the center of the Cu_2_O rod, and the Cu_2_O rods were formed the hollow rods, as shown in Figure 2d. That may be attributed to CuO whiskers arising in the center of the Cu_2_O rods after annealing caused by the stresses between CuO and Cu_2_O. The CuO whiskers that were broken or collapsed during sample handling can be observed from the SEM images.

**Figure 3:**
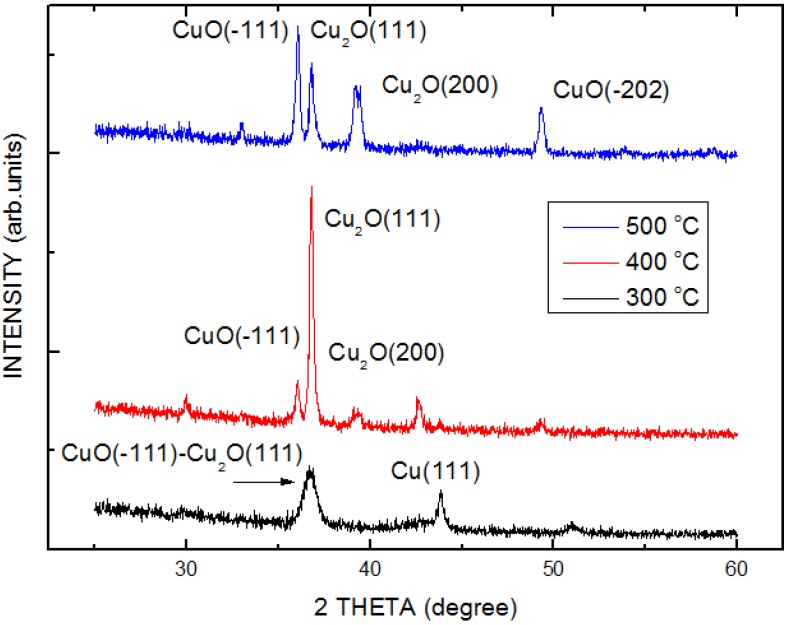
X-ray diffraction patterns of the COC samples with thermal oxidation at various temperatures.

This study employed the X-ray diffraction technique to obtain the main crystalline phases and the possible orientation of crystalline in the films prepared in optimum conditions. Figure 3 shows the XRD spectrum of the measured COC films at various temperatures of 300, 400, and 500 °C. A broad diffraction peak at 36.68° is observed for the sample with thermal treatment at oxidation temperature at 300 °C. It may be attributed to a complex layer consisted of the (−111) plane of the CuO and the (111) plane of the Cu_2_O. A diffraction peak at 43.81° caused by the residue Cu layer is also observed. With oxidation temperature increases from 400 to 500 °C, the broad diffraction peak is separated into two peaks: 36.03° and 36.79°. The diffraction peak at 36.03° corresponding to the (−111) planes of the CuO increases and Cu_2_O peak decreases at 500 °C, and the Cu peak is quenched. This means that the Cu layer was completely depleted and the CuO whiskers were formed. All samples in this study are CuO-Cu_2_O complex layer.

**Figure 4:**
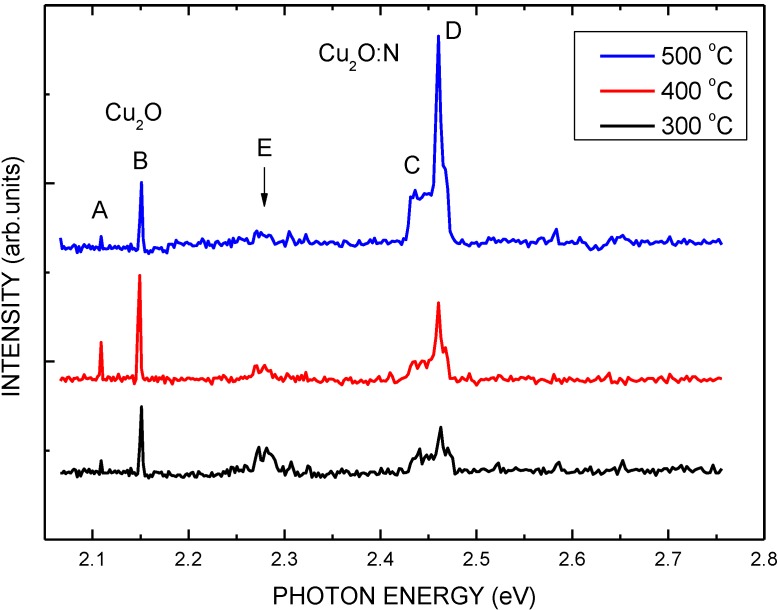
PL spectra of the COC samples with thermal oxidation at various temperatures.

Figure 4 demonstrates the oxidation temperatures dependence of the photoluminescence (PL) spectra of the COC layers. The spectra of the COC layers include four peaks, *i.e.*, A at 2.11 eV, B at 2.15 eV, C at 2.44 eV, and D at 2.46 eV [23]. Peaks A and B are associated with the free exiton and band-to-band transition of Cu_2_O layers, respectively. On the other hand, peaks C and D are associated with the free exiton and band-to-band transition of Cu_2_O:N layers, respectively. The intensity of peaks C and D increase with oxidation temperature increasing. When the Cu layer was in the process of oxidation in ambient air, nitrogen atoms also penetrate into the Cu layer to form the Cu_2_O:N layer. The Cu_2_O:N layer has a higher band gap (2.46 eV) than that of the Cu_2_O layer (2.15 eV) [9]. The values of the band-to-band transition of the layers agree with Reference 9. The PL spectra of the COC layers had a broad yellow band, denoted as E at 2.29 eV. It is may be attributed to deep level caused by defects in the COC layers during oxidation. The intensity of the E band decreases with increasing oxidation temperature. This implies that there is improvement of the crystal quality of the COC layers.

**Figure 5:**
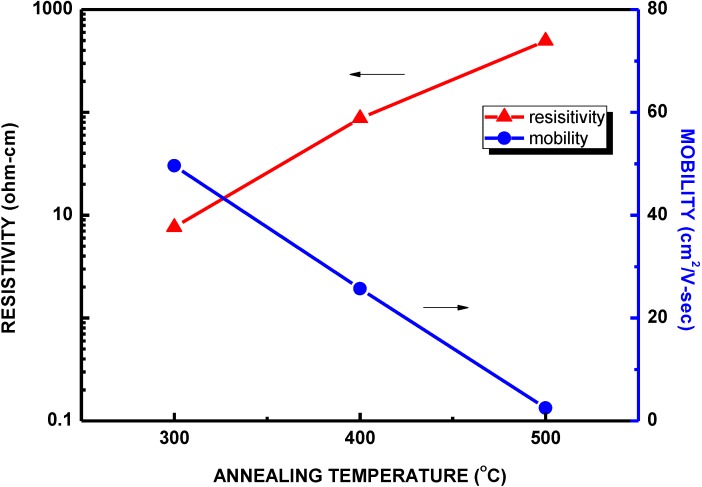
The sheet resistance and the mobility as a function of thermal oxidation annealing temperatures.

Figure 5 plots both the resistivity and the mobility of the COC layers as functions of various oxidation temperatures. As the oxidation temperature increased, the resistivity of COC layers linearly increased to 495 ohm-cm while the mobility declined to 2.5 cm^2^/Vs. The increasing of resistivity and the decreasing of mobility of the COC layers with increasing oxidation temperature may be attributed to the carrier scattering by the ionized acceptors [24,25]. It induced by the incorporation of nitrogen.

**Figure 6:**
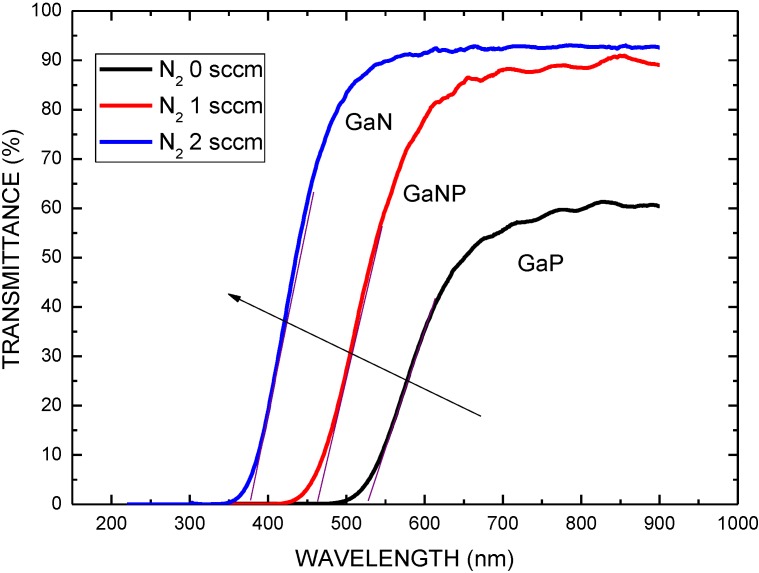
Transmittance spectra of the GaP, GaNP, and GaN layers as a function of nitrogen flow rate.

Sputtered GaP buffer layer with energy gap of 2.24 between the ZnO layer and the COC layer was employed in this study to improve the performance of solar cell structure. Figure 6 plots the transmittance spectra of the Ga(N)P films in the ranges 200 to 900 nm. As shown in Figure 6, the transmittance edge of spectra is to be 370, 460, and 530 nm corresponding to the nitrogen gas flow rate of 0, 1, 2 sccm as sputtering, respectively. As the nitrogen gas flow rate increases from 0 to 2 sccm, the transmittance edge of the spectra demonstrates a blueshift. The blueshift may be attributed to the material transformation from GaP to GaN owing to phosphorous atom replaced by nitrogen atom. This can be attributed to the difference in atom radius. Phosphorous atoms have larger atomic radius (100 pm) than that of nitrogen atoms (65 pm).

**Figure 7:**
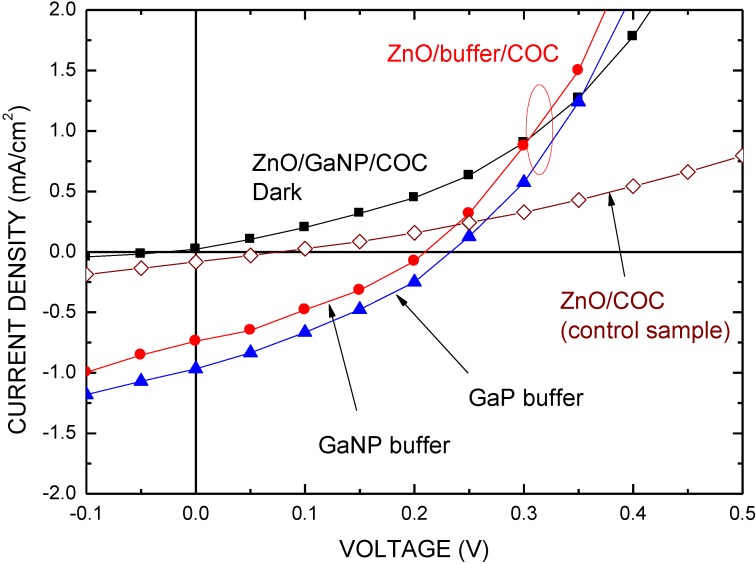
Typical *J*-*V* characteristics of the ZnO/Ga(N)P/COC heterojunction with and without illumination, respectively.

Figure 7 shows the *J*-*V* characteristics of the ZnO/Ga(N)P buffer/COC heterojunction with and without illumination, respectively. The *J*-*V* characteristics of ZnO/COC structure as the control sample are also shown, for comparison. The cell performance was measured under AM 1.5 illumination with a solar intensity of 10 mW/cm^2^ at 25 °C. The cell has an active area of 0.2 × 0.4 cm^2^ and no antireflective coating. Table 1 presents the main characteristics of these devices in this work. As shown in Figure 7, the short current and open voltage can notably improve when the Ga(N)P buffer layer is inserted into the structure. The GaNP has higher electron affinity than that of the GaP. Therefore, the ZnO/GaP/COC structure solar cells exhibited better performance than that of the ZnO/GaNP/COC structure in this study: short circuit current density (*J*_sc_) of 0.968 mA/cm^2^, open circuit voltage (*V*_oc_) of 0.234 V, and fill factor (FF) of 0.316. Therefore, the conversion efficiency (η) can estimated to be 0.72%. The ZnO/COC structure (control sample) demonstrates a low efficiency. This may be attributed to the thermal treatment destroying the ZnO-COC interface because the COC is prone to reduction to Cu at its interface between ZnO and COC, resulting in large interface states and oxygen vacancies [26,27]. Therefore, the benefit of the buffer layer is that it protects the interface during the post-thermal treatment.

**Table 1:** The parameters of different solar cells.

Structure	*V*_oc_ (V)	*J*_sc_ (mA/cm^2^)	FF	η (%)	*R*_s_ (Ω)	*R*_sh_ (Ω)
ZnO/COC	0.077	0.083	0.250	0.016	54	–
ZnO/GaP/COC	0.234	0.968	0.316	0.720	8	61
ZnO/GaNP/COC	0.211	0.738	0.307	0.478	6	53

The series resistance (*R*_s_) in a solar cell is a major parameter, and it is estimated by the relation:

(1) $$\frac{dV}{dI}=R_{s}+\frac{nkT}{q}{\left(I+I_{ph}\right)}^{-1}$$

where *I_ph_* is the photocurrent, and *n*, *k*, and *T* are the ideality factor, Boltzmann constant, and temperature, respectively [28]. According to Figure 7, the series resistance of the control sample, the ZnO/GaP/COC structure, and the ZnO/GaNP/COC structure are 54, 8, and 6 Ω, respectively. The typical value of a silicon solar cell is around 1 Ω. In this work, the main cause of low power conversion efficiency is high series resistance and low shunt resistance [28]. Although the series resistance is reduced by introduction of the sputtered Ga(N)P buffer layer owing to the carrier transition improvement, interconnect resistance of thw interface between the COC layer, the Ga(N)P layer, and ZnO layer is high, as shown in Figure 1a. Another factor is resistance of materials. The sputtered Ga(N)P buffer layer contains a huge oxygen atom incorporated from the sputtering system. The content in the sputtered Ga(N)P is about 0.1% measured by EDX spectrometer. On the other hand, low shunt resistance is caused by the surface states and defects in the materials. The shunt resistance (*R*_sh_) is estimated by the relation:

(2) $$I=I_{0}\times [\text{exp}(q\frac{V-IR_{s}}{nkT})-1]+\frac{V-IR_{s}}{R_{sh}}-I_{ph}$$

where *I*_0_ is the saturation current [29]. The shunt resistance of the ZnO/GaP/COC structure and the ZnO/GaNP/COC structure are 61 and 53 Ω, respectively. Calculating the shunt resistance of the control sample was neglected due to high series resistance. The results are better than that of Reference 28. In order to develop a commercial thin film solar cell with high efficiency, the future is to optimize the thickness, oxidation temperature and ambient temperature of COC, the sputtering and annealing conditions of GaP and the interface between COC and GaP to suppress the surface states and defects in the cells, and to improve the quality of layers and carrier collection.

## 4. Conclusions

ZnO/GaP/COC inverse heterostructures have been prepared by the magnetron sputtering method and oxidation method. The GaP and COC films were used as a buffer and absorber in the cell structure, respectively. The band gap or absorption edge of ZnO, GaP, and COC is 3.37, 2.24, and 2.15 eV, respectively. The role of the GaP buffer layer is to provide a smooth bridge between the ZnO and the COC to improve carrier transition and reduce leakage. The measured parameters of cells were the *J*_sc_, the *V*_oc_, *FF*, and *η*, which had values of 0.968 mA/cm^2^, 0.234 V, 0.316, and 0.72%, respectively, under AM 1.5 illumination without optimum processing parameters. Therefore, as mentioned in the introduction, the GaP layer is more suitable as a buffer layer than the GaNP layer, owing to the energy gap between the ZnO layer and COC layer in the former. However, in order to develop commercial thin film solar cells with high efficiency, further work is needed. For example, the thickness of the COC layer has been shown to be very important in ZnO-COC solar cells [30]. In addition, the interface between COC and GaP also has to be studied.

## References

[1] Noguet C., Tapiero M., Schwab C., Zielinger J.P., Trivich D., Komp R.J., Wang E.Y., Weng K. Cuprous Oxide as a Photovoltaic Converter. Proceedings of the Photovoltaic Solar Energy Conference.

[2] Berezin A.A., Weichman F.L. (1981). Photovoltaic effect in cuprous oxide-copper junctions in relation to the optical absorption spectrum of cuprous oxide. Solid State Commun..

[3] Tapiero M., Noguet C., Zielinger J.P., Schwab C., Pierrat D. (1979). Conversion photovoltaïque dans Cu_2_O. Rev. Phys. Appl..

[4] Olsen L.C., Addis F.W., Bohara R.C. Investigation of Cu_2_O Solar Cells. Proceedings of the 14th IEEE Photovoltaic Specification Conference.

[5] Wang E.Y., Trivich D., Sawaiha H., Thomas G. Cuprous Oxide Schottky Photovoltaic Cells as Potential Solar Energy Converters. Proceedings of the Complex International Conference.

[6] Poizot P., Laruelle S., Grugeon S., Dupont L., Tarascon J.M. (2000). Nano-sized transition-metal oxides as negative-electrode materials for lithium-ion batteries. Nature.

[7] Jeong S.S., Mittiga A., Salza E., Masci A., Passerini S. (2008). Electrodeposited ZnO/Cu_2_O heterojunction solar cells. Electrochim. Acta.

[8] Mittiga A., Salza E., Sarto F., Tucci M., Vasanthi R. (2006). Heterojunction solar cell with 2% efficiency based on a Cu_2_O substrate. Appl. Phys. Lett..

[9] Nakano Y., Saeki S., Morikawa T. (2009). Optical bandgap widening of p-type Cu_2_O films by nitrogen doping. Appl. Phys. Lett..

[10] Tanaka H., Shimakawa T., Miyata T., Satob H., Minami T. (2004). Electrical and optical properties of TCO-Cu_2_O heterojunction devices. Thin Solid Films.

[11] Zhang D.K., Liu Y.C., Liu Y.L., Yang H. (2004). The electrical properties and the interfaces of Cu_2_O/ZnO/ITO p-i-n heterojunction. Phys. B Condens. Matter.

[12] Li Q., Xu M., Fan H., Wang H., Peng B., Long C., Zhai Y. (2013). Electrical charge conductivity behavior of electrodeposited Cu_2_O/ZnO heterojunction thin films on PET flexible substrates by impedance spectroscopy analysis. J. Mater. Sci..

[13] Musselman K.P., Wisnet A., Iza D.C., Hesse H.C., Scheu C., MacManus-Driscoll J.L., Schmidt-Mende L. (2010). Strong efficiency improvements in Ultra-low-Cost inorganic nanowire solar cells. Adv. Mater..

[14] Musselman K.P., Marin A., Schmidt-Mende L., MacManus-Driscoll J.L. (2012). Incompatible length scales in nanostructured Cu_2_O solar cells. Adv. Funct. Mater..

[15] Liu Y., Turley H.K., Tumbleston J.R., Samulski E.T., Lopez R. (2011). Minority carrier transport length of electrodeposited Cu_2_O in ZnO/Cu_2_O heterojunction solar cells. Appl. Phys. Lett..

[16] Fenske F., Kliefoth K., Elstner L., Selle B. (1996). ZnO/c-Si heterojunction interface tuning by interlayers. J. Mater. Sci..

[17] De A., Pryor C.E. (2010). Predicted band structures of III-V semiconductors in the wurtzite phase. Phys. Rev. B.

[18] Belabbes A., Panse C., Furthműller J., Bechstedt F. (2012). Electronic bands of III-V semiconductor polytypes and their alignment. Phys. Rev. B.

[19] Assali S., Zardo I., Plissard S., Kriegner D., Verheijen M.A., Bauer G., Meijerink A., Belabbes A., Bechstedt F., Haverkort J.E.M. (2013). Direct band gap wurtzite gallium phosphide nanowires. Nano Lett..

[20] Park J.H., Natesan K. (1993). Oxidation of copper and electronic transport in copper oxides. Oxid. Met..

[21] Yuan L., Wang Y., Mema R., Zhou G. (2011). Driving force and growth mechanism for spontaneous oxide nanowire formation during the thermal oxidation of metals. Acta Mater..

[22] Wang J.P., Cho W.D. (2009). Oxidation behavior of pure copper in oxygen and/or water vapor at intermediate temperature. ISIJ Int..

[23] Gastev S.V., Kaplyanskii A.A., Sokolov N.S. (1982). Relaxed excitons in Cu_2_O. Solid State Commun..

[24] Akimoto K., Ishizuka S., Yanagita M., Nawa Y., Paul G.K., Sakurai T. (2006). Thin film deposition of Cu_2_O and application for solar cells. Solar Energy.

[25] Zang Z., Nakamura A., Temmyo J. (2013). Single cuprous oxide films synthesized by radical oxidation at low temperature for PV application. Opt. Express.

[26] Olsen L.C., Bohara R.C., Urie M.W. (1979). Explanation for low-efficiency Cu_2_O Schottky-barrier solar cells. Appl. Phys. Lett..

[27] Olsen L.C., Addis F.W., Miller W. (1982). Experimental and theoretical studies of Cu_2_O solar cells. Solar Cells.

[28] Sze S.M., Ng K.K. (2007). Physics of Semiconductor Devices.

[29] Sablon K.A., Little J.W., Sergeev A., Vagidov N., Mitin V. (2012). Solar cell with built-in charge: Experimental studies of diode model parameters. J. Vac. Sci. Technol. A.

[30] Musselman K.P., Marin A., Wisnet A., Scheu C., MacManus-Driscoll J.L., Schmidt-Mende L. (2011). A novel buffering technique for aqueous processing of zinc oxide nanostructures and interfaces, and corresponding improvement of electrodeposited ZnO-Cu_2_O photovoltaics. Adv. Funct. Mater..

